# Evaluation of the Nicotinic Acetylcholine Receptor-Associated Proteome at Baseline and Following Nicotine Exposure in Human and Mouse Cortex

**DOI:** 10.1523/ENEURO.0166-16.2016

**Published:** 2016-08-16

**Authors:** Tristan D. McClure-Begley, Irina Esterlis, Kathryn L. Stone, TuKiet T. Lam, Sharon R. Grady, Christopher M. Colangelo, Jon M. Lindstrom, Michael J. Marks, Marina R. Picciotto

**Affiliations:** 1 Department of Psychiatry, Yale University School of Medicine, New Haven, Connecticut 06508; 2 Institute for Behavioral Genetics, University of Colorado, Boulder, Colorado 80303; 3 Department of Molecular, Cellular and Developmental Biology, University of Colorado, Boulder, Colorado 80309; 4 W.M. Keck Biotechnology Resource Laboratory, Yale University School Medicine, New Haven, Connecticut 06509; 5 Department of Molecular Biophysics & Biochemistry, Yale University, New Haven, Connecticut 06520; 6 Department of Neuroscience, Medical School of the University of Pennsylvania, Philadelphia, Pennsylvania 19104; 7 Department of Psychology and Neuroscience, University of Colorado, Boulder, Colorado 80309

**Keywords:** addiction, cortex, depression, nicotine, nicotinic acetylcholine receptors, proteomics

## Abstract

Nicotinic acetylcholine receptors (nAChRs) support the initiation and maintenance of smoking, but the long-term changes occurring in the protein complex as a result of smoking and the nicotine in tobacco are not known. Human studies and animal models have also demonstrated that increasing cholinergic tone increases behaviors related to depression, suggesting that the nAChR-associated proteome could be altered in individuals with mood disorders. We therefore immunopurified nAChRs and associated proteins for quantitative proteomic assessment of changes in protein–protein interactions of high-affinity nAChRs containing the β2 subunit (β2*-nAChRs) from either cortex of mice treated with saline or nicotine, or postmortem human temporal cortex tissue from tobacco-exposed and nonexposed individuals, with a further comparison of diagnosed mood disorder to control subjects. We observed significant effects of nicotine exposure on the β2*-nAChR-associated proteome in human and mouse cortex, particularly in the abundance of the nAChR subunits themselves, as well as putative interacting proteins that make up core components of neuronal excitability (Na/K ATPase subunits), presynaptic neurotransmitter release (syntaxins, SNAP25, synaptotagmin), and a member of a known nAChR protein chaperone family (14-3-3ζ). These findings identify candidate-signaling proteins that could mediate changes in cholinergic signaling via nicotine or tobacco use. Further analysis of identified proteins will determine whether these interactions are essential for primary function of nAChRs at presynaptic terminals. The identification of differences in the nAChR-associated proteome and downstream signaling in subjects with various mood disorders may also identify novel etiological mechanisms and reveal new treatment targets.

## Significance Statement

Nicotinic acetylcholine receptors (nAChRs) are ligand-gated ion channels responsible for rapid excitatory acetylcholine signaling, which modulate numerous circuits involved in complex behaviors. In addition, nAChRs are the primary targets for the reinforcing effects of nicotine in tobacco products. Proteins associated with nAChRs are critical for receptor trafficking, localization, and function. Identifying the core elements of the nAChR-associated proteome that are conserved across human and mouse brain is essential for understanding how these receptors are regulated, and how their functions and interactions are altered by long-term exposure to nicotine.

## Introduction

Individuals who smoke have significantly higher numbers of high-affinity nicotinic acetylcholine receptors (nAChRs) compared to nonsmokers, as measured in both postmortem brain studies (Breese et al., 1997) and SPECT imaging studies with a radiotracer specific for nAChRs containing the β2 subunit (β2*-nAChRs; Staley et al., 2006). This upregulation appears to be due primarily to the nicotine in tobacco, since rodent studies demonstrate that long-term exposure to nicotine alone results in a similar upregulation of β2*-nAChRs in brain (Marks et al., 1992). While this change in receptor number can alter signaling through nAChRs (Buisson and Bertrand, 2001; Govind et al., 2012), the effect of nicotine treatment and upregulation on the associated proteome is not known. Identifying the complex associated with upregulated nAChRs would be helpful in understanding both the basic biology of the nAChR system, as well as the consequences of smoking on nAChR-induced intracellular signaling. Comparison of the nAChR-associated proteome in human smokers versus rodents exposed to nicotine is also important to identify molecular changes selective for nicotine alone, rather than the ∼4000 other constituents of tobacco smoke. Further, the identification of the changes in the nAChR-associated proteome as a result of nicotine exposure could provide insights into the mechanisms underlying nAChR upregulation, as well as determining whether nicotine-mediated upregulation alters the downstream signaling of these receptors. Finally, comparison of the human and rodent nAChR-associated proteome at baseline could identify conserved signaling pathways that important for ongoing smoking behavior and other disorders associated with changes in nAChR signaling.

Among disorders associated with smoking, mood disorders (MDs) are of particular interest because of the high comorbidity reported (Lerman et al., 1996; Laje et al., 2001; Mineur and Picciotto, 2009) and because several lines of evidence implicate the dysfunction of the cholinergic system in depressive states (for review, see Mineur and Picciotto, 2010; Philip et al., 2010). Animal and human studies suggest that increased ACh activity can induce symptoms of depression, whereas decreased cholinergic activity may be associated with mania (Dilsaver, 1986; Dilsaver and Coffman, 1989). Consistent with challenge studies using a cholinesterase inhibitor to increase nAChR occupancy (Esterlis et al., 2013), imaging studies suggest that brain ACh levels may be higher in individuals with major depressive or bipolar disorder when they are actively depressed (Saricicek et al., 2012; Hannestad et al., 2013). With respect to nAChRs, decreasing the activity of β2*-nAChRs using molecular genetics, pharmacological antagonism, or partial agonism can alter behavior in tests of antidepressant efficacy (Caldarone et al., 2004; Rabenstein et al., 2006; Mineur et al., 2007, 2009). Overall nAChR signaling may be altered in individuals with depressive disorders, suggesting that it could be important to identify any potential differences in the functional regulation of β2*-nAChRs and protein–protein interactions in patients with affective disorders.

In the current study, we took a dual approach to identify changes in the nAChR-associated proteome with nicotine exposure and to determine whether any changes were also apparent in individuals with a mood disorder. We obtained postmortem tissue from the temporal cortex of human subjects and used tissue cotinine content as a determinant of tobacco use to determine whether there was any difference in the nAChR-associated proteome as a function of receptor number. (Note that we use “tobacco use” rather than “smoker” since it is not possible to discriminate between modes of tobacco use from brain metabolite levels). Among the human subjects, we evaluated cortical tissue from both healthy control subjects and matched individuals with a mood disorder diagnosis to identify any differences in receptor number or response to smoking. In addition, we exposed mice with differing levels of high-affinity nAChRs to nicotine in the long term to identify changes in the associated proteome that do or do not scale with nAChR number to determine which smoking-induced changes in human tissue can be attributed to the nicotine in tobacco. These results are the first to compare human and mouse nAChR-associated proteins, and the findings identify conserved proteins in the β2*-nAChR interactome and the effects of nicotine exposure that are valid to explore with other models.

## Materials and Methods

### Human temporal cortex tissue collection

Human temporal cortex samples were obtained from the Douglas Brain Bank as frozen tissue sections, ranging from 0.29 to 1.03 g wet weight of tissue. Visual inspection of each section indicated approximately equivalent amounts of gray and white matter per section. All tissue samples were numbered by the supplier, with diagnosis (major depressive, bipolar, mood disorder not otherwise specified, or no mood disorder) and cause of death as the only additional information. Experimental results were not sorted by diagnosis until after results were obtained; experimenters were blind to the groups during data collection.

### Mice

Wild-type mice and mice carrying genetic deletion of the β2 nAChR subunit (Picciotto et al., 1995, 1998) or the α4 nAChR subunit (Ross et al., 2000) were used for these studies. The animals used were wild type (β2^+/+^), heterozygous (β2^+/−^), α4/β2 double-heterozygous (α4^+/−^β2^+/−^), and knock-out (β2^−/−^) mice. Mice were bred and maintained in the animal colonies at the University of Colorado and Yale University. Female mice were used for this study because females are more resilient to chronic nicotine administration (Hatchell and Collins, 1980) and were housed in groups of no more than five same-sex littermates per cage, on a 12 h light/dark cycle with *ad libitum* access to food and water. All protocols involving the use of live animals were approved by and followed the guidelines of the institutional animal care and use committees of the University of Colorado and Yale University.

### Surgery and long-term nicotine treatment

Mice were fitted with jugular catheters essentially as described previously (Marks et al., 2011), with minor modifications. Briefly, mice were anesthetized with an intraperitoneal injection of ketamine/xylazine (100/8 mg/kg) and a small incision (<1 cm) was made above the right clavicle to access the superficial aspect of the jugular vein. A small catheter (inner diameter, 0.51 mm; outer diameter, 0.94 mm) made of medical-grade silastic tubing and filled with sterile saline solution was inserted into the exposed vein and anchored in position with two surgical silk sutures. The catheter was passed under the skin through a second small (<1 cm) incision made in the midthoracic region of the skin and was attached to a stainless steel post anchored under the skin with veterinary adhesive. Incisions were cleaned with 0.1% iodine in ethanol and closed with veterinary adhesive. Mice were given an intraperitoneal injection of buprenorphine (0.1 mg/kg) following surgery and were allowed to recover in warm (37^°^C) individual cages. For long-term infusion, mice were housed individually, and the jugular catheter was attached to a 1 ml syringe pump and infused with saline at a rate of 35 μl/h for 48 h. Vehicle control mice received sterile saline, and nicotine-treated animals received 4 mg/kg/h nicotine (free base, neutralized with HCl) for a total of 10 d. On the last day of nicotine treatment, mice were detached from the syringe pump and allowed to clear the nicotine for 2 h. Mice were killed by cervical dislocation, and the brain was rapidly removed and placed on an ice-cold surface for dissection. Each brain was dissected, and the cortex was frozen at −80^°^C until analysis.

### [^125^I]-Epibatidine binding

Quantification of high-affinity nAChR binding sites in brain tissue was performed essentially as described previously (Hannestad et al., 2013) with minor modifications. Briefly, a small prism of tissue was removed from the large frozen section by dissection on an ice-cold surface with a sterile scalpel. The excised tissue was placed immediately in 0.25 ml ice-cold 0.1× phosphate buffer (PB; 12.8 mm NaCl, 0.24 mm KCl, 0.32 mm CaCl_2_, 0.12 mm KH_2_PO_4_, 0.12 mm MgSO_4_.7H_2_O, 2.5 mm HEPES hemi-Na, pH7.5). Samples were homogenized by hand in a glass/Teflon tissue grinder and diluted to a final volume of 0.5 ml in 0.1× PB. The tissue homogenate was centrifuged at 10,000 x g at 4^°^C for 20 min, and the supernatant was discarded. The tissue pellet was resuspended in 0.5 ml of 0.1× PB and centrifuged for 20 min at 10,000 × *g* at 4^°^C. This wash step was repeated a total of three times in order to ensure the removal of substances that could compete with [^125^]I-epibatidine binding (i.e., nicotine, ACh). For ligand binding to particulate fractions, 10 μl aliquots of tissue homogenate were added to wells of 96-well plates containing 200 pm [^125^I]-epibatidine as the final concentration (Whiteaker et al., 2000). Nonspecific binding was assessed in the presence of 100 μm cytisine. The protein content of each sample was determined using the method of Lowry et al. (1951).

### Immunopurification of β2*-nAChRs

Purification of β2*-nAChRs with mAb295-coupled M270 Dynabeads from mouse and human cortical tissue samples was conducted essentially as described previously (McClure-Begley et al., 2013), with minor modifications. For the mouse cortex samples, dissected cortices (both hemispheres) from two mice were pooled to create one homogenate for immunoprecipitation (IP); human samples were used as is, and the volume of homogenization buffer was adjusted to equal 4 volumes based on the total wet weight of the tissue. Briefly, brain tissue sections were homogenized by hand with 17 strokes in a glass/Teflon tissue grinder with 4 volumes of ice-cold PBS (pH 8.0), and placed on ice. To each tissue homogenate, dithiobis[succinimidyl propionate] [DSP (also called Lamont's Reagent); 12 Å linker span; 10 mm in DMSO] was added to a final concentration of 1 mm to stabilize labile interactions prior to detergent extraction. Tissue homogenates were incubated on ice for 45 min with DSP prior to the addition of Tris to a final concentration of 10 mm to quench the crosslinking reaction. Samples were centrifuged at 10,000 × *g* at 4^°^C for 20 min, and the supernatant was collected and frozen at −20^°^C, and the pellet was washed by resuspension in TBS before being centrifuged again. The washed pellet was resuspended in 2 ml of extraction buffer (EB; in mm: 121.9 NaCl, 2.68 KCl, 10.14 Na_2_HPO_4_, 1.76 KH_2_PO_4_, 5 EDTA, 5 EGTA, 5 NaF, 0.1 Na_3_VO_4_, 1.0 PMSF, 10 μg/ml each of aprotonin, prepstatin A, leupeptin, 0.and 6% Triton X-100, pH 7.4) and incubated for 30 min with gentle rotation at room temperature. The extract was clarified by centrifugation at 10,000 × *g* at 4^°^C for 20 min, and the supernatant was collected. A 100 μl aliquot of clarified extract was taken at this point to quantify total extracted nAChR content with [^125^I]-epibatidine binding, as described below. The remaining extract was added to 5 ml of polystyrene culture tubes containing a volume of mAb295-Dynabeads equivalent to 50% of the input volume and incubated with gentle rotation at 4^°^C overnight. The following morning, sample tubes were placed on a magnetic stand to separate the mAb295 beads, and a 100 μl aliquot of the supernatant was collected to assess nAChR capture efficiency. For the quantification of nAChR immunopurification, 20 μl aliquots of input and post-IP tissue lysate were labeled with 200 pm [^125^I]-epibatidine overnight at 4^°^C, and protein precipitated by 50 μl of 40% w/v polyethylene glycol (PEG) added to each well. PEG-precipitated binding sites were captured on filter membranes as for the particulate binding studies. The depletion of binding sites was used to estimate receptor content of each sample post-elution.

### Sample preparation and protein identification by liquid chromatography-tandem mass spectrometry

Dynabeads carrying mAb295-captured nAChRs and bound proteins were washed two times with 1 ml of PBS (137 mm NaCl, 27 mm KCl, 100 mm Na_2_HPO4, 18 mm KH_2_PO_4_, pH 7.5) containing 0.01% Tween-20, and one time with PBS prior to elution with 0.5 ml of 0.1 m NH_4_OH and 1 mm EDTA, pH 10.0. Elution was performed twice, and eluates were pooled prior to lyophilization in a Thermo Savant SPD1010 Speedvac. Eluted proteins were reduced, alkylated, and digested as described previously (McClure-Begley et al., 2013). Mouse cortex samples were labeled in an eight-plex iTRAQ labeling scheme, and peptide sequencing was performed on an ABSciex 5600 triple- time-of-flight mass spectrometer, with iTRAQ quantitation in ProteinPilot software, as has been described (McClure-Begley et al., 2013). For the human temporal cortex samples, the eluted protein pellet was redissolved in 30 µl of 8 m urea/0.4 m ammonium bicarbonate, pH 8.0. The addition of 3 µl of 45 mm DTT was followed by vortexing, and samples were then incubated at 37°C for 20 min. Reduced samples were cooled to room temperature and alkylated with 3 µl of 100 mm iodoacetamide for 20 min in the dark. Water (74 µl) was added to dilute the sample prior to trypsin digestion (10 µl, 0.5 mg/ml) at 37°C overnight. Samples were desalted on a Macrospin cartridge (Nest Group), and eluent was dried in a Speedvac. The pellet was resuspended in 10 µl of 70% formic acid and 40 µl of 50 mm sodium PB. Equal peptide amounts were aliquoted into glass vials for injection.

Proteomic analysis was performed using a label-free quantification (LFQ) method on an Orbitrap Elite equipped with a Waters Symmetry C18 (180 µm × 20 mm) trap column and a 1.7 µm, 75 µm × 250 mm nanoAcquity ultra high-performance liquid chromatography (UPLC) column (35°C). Trapping was performed using 99% Buffer A (99.9% water, 0.1% formic acid) and peptide separation with a linear gradient of solvent A (0.1% formic acid in water) and solvent B (0.075% formic acid in acetonitrile) over 90 min, at a flow rate of 300 nl/min. MS spectra were acquired in the Orbitrap using a 1 µm scan and a maximum injection time of 900 ms followed by three data-dependant MS/MS acquisitions in the ion trap (with precursor ions threshold of >3000). The total cycle time for both MS and MS/MS acquisition was 2.4 s. Peaks targeted for MS/MS fragmentation by collision-induced dissociation were first isolated with a 2 Da window followed by normalized collision energy of 35%. Dynamic exclusion was activated where former target ions were excluded for 30 s.

Feature extraction, chromatographic/spectral alignment, data filtering, and statistical analysis were performed using Nonlinear Dynamics Progenesis LC-MS software (www.nonlinear.com). The raw data files were imported into the program, a sample run was chosen as a reference (usually at or near the middle of all runs in a set), and all other runs were automatically aligned to that run in order to minimize retention time (RT) variability between runs. All runs were selected for the detection of peptides with an automatic detection limit for signal-to-noise ratio of 3. Features within RT ranges of 0–25 min and 110–120 min were filtered out (these are outside the range of the peptide elution profile), as were features with a charge of ≥7. A normalization factor was then calculated for each run to account for differences in sample load between injections. The experimental design was set up to group multiple injections from each run. The algorithm then calculated and tabulated the raw and normalized abundances, the maximum fold changes and the ANOVA values for each feature in the dataset. The corresponding MS and MS/MS spectra for the features were exported as a Mascot generic file (.mgf) for database searching to perform protein identification. The .mgf files created by the Progenesis LCMS were searched in-house using the Mascot Search Algorithm (version 2.2.0). The data were searched using the Swiss protein database along with the following search parameters: enzyme, trypsin; variable modifications, carbamidomethyl (Cys), oxidation (Met), and alkylated DSP crosslinker-modified lysine (C_10_H_19_N_2_O_3_S_2_); peptide mass tolerance, ±20 ppm; fragment mass tolerance, ±0.6 Da; charge, +7; maximum missed cleavages, 3; decoy, yes; and instrument type, ESI-TRAP. The resulting search was exported as an .xml file and read into the Progenesis LC-MS software, where search hits, with at least 95% confidence, were matched to corresponding features (precursor ions and abundances). Features/peptides with nonconflicting identification match were used to provide relative quantification of protein expression. The identification of peptides/proteins and quantitative values across the various comparisons were exported as a .csv file, and further downstream data analyses were performed. The means of normalized abundances for proteins identified in three technical replicates for each tissue sample were used in subsequent analyses.

### Cotinine determination from brain tissue lysate by liquid chromatography-tandem mass spectrometry

Following detergent extraction from tissue homogenates and immunopurification of β2*-nAChR complexes with mAb295-coupled Dynabeads, the clarified lysate was frozen at −20^°^C. The lysate was thawed, and a 200 μl aliquot of each sample was subjected to organic extraction according to published methods (Massadeh et al., 2009) with minor modification. Briefly, the clarified tissue lysate was alkalized with 50 μl of 1 m NaOH and mixed with 2.5 ml of 1:1 dichloromethane/hexanes in a glass test tube and vortexed vigorously. The mixture was centrifuged for 10 min at 5000 × *g* at room temperature, and the upper organic layer was removed and placed in a clean borosilicate glass tube, then dried under a gentle stream of nitrogen at room temperature. Samples were reconstituted in 100 μl of 0.1% formic acid in water, and 10 μl of each sample was used per injection for analysis.

All liquid chromatography-tandem mass spectrometry (LC-MS/MS) identifications of cotinine were performed on an ABSciex 4000 QTrap Mass Spectrometer in positive ion mode coupled to a PerkinElmer Flexar FX-15 UPLC unit. For chromatography, solvent A was 99.9% water and 0.1% formic acid, and solvent B was 99.9% acetonitrile and 0.1% formic acid. The elution gradient was as follows: 98% solvent A, 2% solvent B from 0 to 1.8 min; 98% solvent B, 2% solvent A from 1.8 to 2.8 min; and 98% solvent A, 2% solvent B from 2.9 to 6.0 min. A selected reaction monitoring (SRM) method was developed for the quantification of cotinine present in the sample using an isotopically coded analytical standard (d-cotinine; Cerilliant Corporation). As described previously (Jacob et al., 2011), the cotinine parent ion had a mass-to-charge ratio (*m*/*z*) of 177.12 at charge +1 and yielded a specific transition with major product ions of an *m*/*z* values of 80.03 and 98.02. Standard curves showed that intensities of both major product ions were linear with respect to injected cotinine >9.5 ng/μl. Cotinine abundances for each transition from replicate injections were averaged and normalized to the total wet weight of each tissue sample in order to yield units of nanograms per gram tissue to determine nicotine use status.

### Data analysis

SigmaPlot 13.0 and SPSS 23 (2015) were used for data organization and statistical analysis. All ligand binding data are reported in specific femtomoles/milligram protein. For proteins identified with LC-MS/MS, we took the sum of the normalized protein abundance for all proteins scoring above the 95% false discovery rate (FDR) cutoff from three technical replicates per sample and generated arithmetic means that were then log2 transformed to yield a continuous variable with a normal distribution (Smits et al., 2013). Linear regression analysis was performed using a Pearson’s product moment correlation coefficient (*r*) as an indicator of significant positive correlations. Normalization using the calculated abundance of the β2 nAChR subunit (target of the IP) in each sample as the denominator was performed, and ratios relative to control conditions were compared to assess alterations in associated proteins per unit of nAChR.

Mouse cortex iTRAQ data were processed as has been described previously (McClure-Begley et al., 2013). Briefly, mean values of the reporter ion peak areas for every protein identified above the FDR cutoff were divided by the mean value for the control condition (β2^+/+^, saline) for each set and then log2 transformed to yield normalized protein expression ratios. Significant effects of nicotine exposure and mouse genotype (for the mouse samples) or mood disorder diagnosis and nicotine use (human samples) were determined with ANOVA, Holm–Sidak test, or Student’s *t* test, where appropriate as determined by the nature of the experiment, with a 95% confidence interval. A general linear model (GLM) split by protein identification with multiple univariate comparisons was used to determine whether levels of individual human brain proteins [a normally distributed (log2 fold change) continuous response variable] that vary over multiple categorical predictor variables (nicotine, MD, both, or neither) differed in representation compared with the control condition (where the intercept is the mean of the no-nicotine exposure, no-MD diagnosis values). This was only possible for human cortical samples that were run using LFQ, in which there is a discrete interval measurement for every group. Mouse samples were run on iTRAQ in which every eight-plex experiment uses one channel as the control value, which is convoluted into the variance of the other seven reporter ion channels. Thus, the mouse GLM does not include a discrete value for the control group, since reintroducing the control group drags the regression to the origin and biases the estimates of significance.

Data are reported as mean ± SEM, with error terms specifically reported for each value.

## Results

### Effects of smoking and mood disorder diagnosis on nAChR number measured with [^125^I]-epibatidine binding

**Figure 1.**
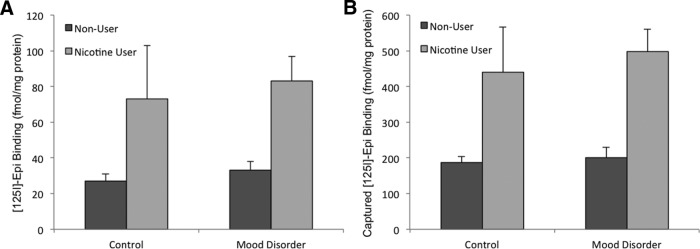
Effects of nicotine use and mood disorder on total high-affinity [^125^I]-epibatidine binding in cell membranes from human temporal cortex samples. ***A***, Nicotine use, determined by tissue cotinine content, has a significant effect on the number of total [^125^I]-epibatidine binding sites (two-way ANOVA with Holm–Sidak multiple comparisons: *F*_(1,17)_ = 9.794, *p* = 0.007), while mood disorder does not have an appreciable effect on the expression of high-affinity nAChRs in the cortex (*F*_(1,17)_ = 0.224, *p* = 0.673). ***B***, β2*-nAChR capture was evaluated by [^125^I]-epibatidine binding following immunoprecipitation from human temporal cortex using mAb295-M270 Dynabeads. Error bars represent standard error of the mean.

We measured high-affinity [^125^I]-epibatidine binding to particulate fractions in order to determine any effects of smoking status and mood disorder diagnosis on nAChR number in human temporal cortex. High levels of α4β2*-nAChRs, the subtype upregulated by concentrations of nicotine typically encountered by smokers (Teaktong et al., 2004), are found in mammalian cortex (Zoli et al., 1998). Smoking status was not included with human tissue samples, so in order to group samples appropriately, we examined the cotinine content of each sample using an SRM LC-MS/MS method with a deuterated cotinine standard. Samples were grouped according to cotinine content and α4β2*-nAChR expression, with “nicotine users” considered as those samples with >15 ng cotinine/g wet tissue weight. Cotinine levels present in tissue did not correlate linearly with [^125^I]-epibatidine binding sites (*r* ^2^ = 0.33), indicating that the postmortem tissue content of the major nicotine metabolite is useful principally for ordinal assignment of nicotine use (nicotine user or nonuser), and cannot be used to further discriminate “heavy/moderate/light” nicotine users. Based on these criteria, there were nine nicotine users and nine nonusers. Consistent with the high comorbidity between smoking and depression, the incidence of nicotine exposure was higher in individuals with a diagnosed mood disorder (six of nine were nicotine users) compared with individuals with no psychiatric diagnosis (three of nine were nicotine users). We observed a significant effect of cotinine level, our empirically determined tobacco use criterion, on nAChR number, as measured by epibatidine binding in homogenates (two-way ANOVA with Holm–Sidak multiple comparisons: *F*_(1,17)_ = 9.794, *p* = 0.007). Analysis revealed no significant effect of mood disorder diagnosis on nAChR number (*F*_(1,17)_ = 0.224, *p* = 0.673) or an interaction of nicotine × mood disorder (*F*_(1,17)_ = 0.04, *p* = 0.836) measured by equilibrium ligand binding (Fig. 1*A*), which is in agreement with previous assessments of nAChR number in postmortem human cortical tissue from individuals with major depression or bipolar disorder (Saricicek et al., 2012; Hannestad et al., 2013).

### Immunoprecipitation of β2*-nAChRs from human temporal cortex and identification of specific interacting proteins by tandem mass spectrometry

Following the extraction of nAChR complexes from human brain tissue, we quantified specific mAb295 immunocapture of β2*-nAChRs by measuring total [^125^I]-epibatidine binding sites in the solubilized tissue extract before and after incubation with mAb295-Dynabeads. In human temporal cortex samples, the recovery of β2*-nAChRs using mAb295-Dynabeads was 74.5 ± 7.6% of input across all samples, with no effect of either smoking status or mood disorder diagnosis (*t* test, *p* = 0.32, *t* = −1.017, df = 16). In mouse cortex samples, despite fractional differences in the percentage of upregulation induced by long-term nicotine exposure as a function of genotype, capture efficiency was 60.9 ± 3.1% of input across the samples, with no significant difference in capture efficiency between the saline control and groups receiving long-term nicotine treatment (*t* test, *p* = 0.06, *t* = −2.108, df = 10), indicating that the immobilized mAb295 performed equally well across the samples regardless of differences in nAChR abundance. As expected based on epibatidine binding levels in homogenates and previously published studies, we observed a significant effect of nicotine use on the amount of β2*-nAChR present in each sample (Fig. 1*B*); however, no significant effect of mood disorder diagnosis or nicotine × mood disorder interaction was identified (two-way ANOVA: main effect, nicotine use: *F*_(1,17)_ = 18.002, *p* < 0.0009; main effect, mood disorder: *F*_(1,17)_ = 0.236, *p* = 0.634; interaction: *F*_(1,17)_ = 0.06, *p* = 0.813).

**Figure 2.**
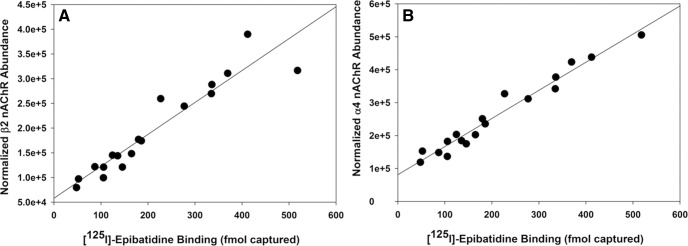
[^125^I]-epibatidine binding to α4β2*-nAChRs in human temporal cortex homogenates correlates well with β2-nAChR (***A***) and α4-nAChR (***B***) subunit abundance estimated by normalized unique peptide abundance determined by peptide LC-MS/MS.

Transgenic mice lacking expression of α4 and β2 nAChR subunits have been used previously to establish a gene dose-dependent linear relationship between α4β2*-nAChRs and their putative associated proteins from whole mouse brain samples using an immunocapture protocol with iTRAQ-labeled peptides for quantitative proteomics (McClure-Begley et al., 2013). Such an approach used nAChR subunit heterozygous and knock-out mice to vary the abundance of nAChRs reliably and to examine relative changes in associated proteins with linear regressions to enhance confidence in identifying interacting proteins. In the human tissue samples used in this study, we observed significant variation in the total nAChR content of each sample, partially due to inherent variability, but also because of the impact of nicotine use. The measured abundance of the β2 nAChR subunit (target of the IP) as well as the abundance of α4 nAChR subunits measured by LFQ proteomics correlate extremely well with the calculated nAChR content of each sample obtained by the depletion of specific [^125^I]-epibatidine binding from tissue extracts (femtomoles vs β2-LFQ: *r* ^2^ = 0.89; femtomoles vs α4-LFQ: *r* ^2^ = 0.96; Fig. 2*A*,*B*).

We identified 118 putative interacting proteins from the abundance estimates provided by proteomic analysis of mAb295 immunopurified β2*-nAChRs from human temporal cortex. We excluded obvious contaminants (epidermal keratins, hornerin, hemoglobins, histones, cytochromes) and any protein with fewer than two unique peptides identified at a false discovery rate of 5%. With these criteria applied, we identified and quantified 78 proteins that coimmunoprecipitated with β2*-nAChRs from human postmortem temporal cortex (Table 1). Of these, 42 proteins were identified as nAChR-interacting proteins in mouse cortex, and an additional 14 were isoforms of identified mouse cortex interacting proteins. An overlapping set of 36 proteins were identified in whole mouse brain as nAChR-interacting proteins, with an additional 17 isoforms of previously identified interacting proteins (McClure-Begley et al., 2013). Further, 33 of 78 proteins identified showed statistically significant correlations of protein abundance with β2-nAChR subunit abundance (Table 2), indicating a likely specific protein–protein interaction or physical proximity at the time of receptor extraction (Table 3). Functional annotation clustering of these 33 proteins using the DAVID Bioinformatics Database analysis (Huang da et al., 2009) showed a significant enrichment for synaptic pathways and neuronal projection (enrichment score, 5.47). Therefore, stable receptor interactions that survive tissue solubilization are likely assembled β2*-nAChRs, and a subset of these nAChRs may be resident in the plasma membrane of neuronal synapses. The identification of a 14-3-3 isoform previously shown to mediate α3*nAChR trafficking to postsynaptic structures (Rosenberg et al., 2008) indicates that some β2* interactions are mediated on both sides of CNS synapses by this family of adaptor proteins. Based on the current dataset, it is possible that some of the captured β2*-nAChRs reside in the postsynaptic plasma membrane, but we failed to identify additional known postsynaptic density components or associated proteins with functional annotations placing them in that neuronal subcellular compartment, so additional experiments will be required to address variations in postsynaptic versus presynaptic β2*-nAChR-interacting proteins.

**Table 1.** Tabulation of all proteins identified from mAb295-immunoisolated β2*-nAChRs from human temporal cortex, indicating which proteins have been identified in mouse cortex or whole mouse brain (Iso, isoform of protein identified; GN, gene name)

Accession_HUMAN	Description	Mouse Ctx	Mouse Brain
1433E	14-3-3 protein ε GN = YWHAE	Iso	Iso
1433G;1433F	14-3-3 protein γ GN = YWHAG	Iso	Iso
1433S	14-3-3 protein σ GN = SFN	Iso	Iso
1433Z	14-3-3 protein ζ/δ GN = YWHAZ	Iso	Yes
ACHA4;ACHA2;ACHA6	Neuronal acetylcholine receptor subunit α4 GN = CHRNA4	Yes	Yes
ACHB2	Neuronal acetylcholine receptor subunit β2 GN = CHRNB2	Yes	Yes
ACTB;ACTBM;POTEE	Actin, cytoplasmic 1 GN = ACTB	Yes	Iso
ACTC;ACTA	Actin, α cardiac muscle 1 GN = ACTC1	Iso	Yes
ACTN1;ACTN3	α-Actinin-1 GN = ACTN1	No	Yes
ACTN2	α-Actinin-2 GN = ACTN2	No	Yes
ACTN4	α-Actinin-4 GN = ACTN4	No	Iso
ADDA	α-Adducin GN = ADD1	No	No
ADT1;ADT2;ADT4	ADP/ATP translocase 1 GN = SLC25A4	Yes	Yes
ALBU	Serum albumin GN = ALB	No	No
ANK2	Ankyrin-2 GN = ANK2	No	Yes
AP1B1	AP-1 complex subunit β1 GN = AP1B1	No	No
ARP2	Actin-related protein 2 GN = ACTR2	No	Iso
ARP3	Actin-related protein 3 GN = ACTR3	No	Yes
ARPC3	Actin-related protein 2/3 complex subunit 3 GN = ARPC3	No	Iso
ARPC4	Actin-related protein 2/3 complex subunit 4 GN = ARPC4	No	Iso
AT1A1;AT12A	Sodium/potassium-transporting ATPase subunit α1 GN = ATP1A1	Iso	Iso
AT1A2	Sodium/potassium-transporting ATPase subunit α2 GN = ATP1A2	Iso	Yes
AT1A3;AT1A4;ATP4A	Sodium/potassium-transporting ATPase subunit α3 GN = ATP1A3	Yes	Yes
AT1B1	Sodium/potassium-transporting ATPase subunit β1 GN = ATP1B1	Yes	Yes
AT2B2;AT2B1	Plasma membrane calcium-transporting ATPase 2 GN = ATP2B2	No	No
ATPA	ATP synthase subunit α, mito GN = ATP5A1	Yes	Yes
ATPB	ATP synthase subunit β, mito GN = ATP5B	Yes	Yes
BASP1	Brain acid-soluble protein 1 GN = BASP1	No	No
CALM	Calmodulin GN = CALM1	Yes	Yes
CANB1	Calcineurin subunit B type 1 GN = PPP3R1	No	No
CLH1	Clathrin heavy chain 1 GN = CLTC SV = 5	Yes	No
CNTN1	Contactin-1 GN = CNTN1	No	Yes
COX2	Cytochrome c oxidase subunit 2 GN = MT-CO2	No	Yes
COX41	Cytochrome c oxidase subunit 4 isoform 1, mito GN = COX4I1	No	Yes
COX6C	Cytochrome c oxidase subunit 6C GN = COX6C	No	Yes
CX6B1	Cytochrome c oxidase subunit 6B1 GN = COX6B1	No	Yes
CX7A2	Cytochrome c oxidase subunit 7A2, mito GN = COX7A2	No	Yes
DREB	Drebrin GN = DBN1	No	Yes
DYN1	Dynamin-1 GN = DNM1	No	No
E41L3	Band 4.1-like protein 3 GN = EPB41L3	No	No
EAA1	Excitatory amino acid transporter 1 GN = SLC1A3	Iso	Iso
EAA2	Excitatory amino acid transporter 2 GN = SLC1A2	Yes	Yes
EF1A1;EF1A2	Elongation factor 1-α1 GN = EEF1A1	No	No
FCGBP	IgGFc-binding protein GN = FCGBP	No	No
FRIH	Ferritin heavy chain GN = FTH1	No	No
FRIL	Ferritin light chain GN = FTL	No	No
G3P	Glyceraldehyde-3-phosphate dehydrogenase GN = GAPDH	Yes	Yes
GBB1;GBB2;GBB3;GBB4	Guanine nucleotide-binding protein G(I)/G(S)/G(T) subunit β1 GN = GNB1	Yes	Yes
GELS	Gelsolin GN = GSN	No	Yes
GFAP	Glial fibrillary acidic protein GN = GFAP	Yes	Yes
GNAO;GNA12	Guanine nucleotide-binding protein G(o) subunit α GN = GNAO1	Yes	Yes
GPM6A	Neuronal membrane glycoprotein M6-a GN = GPM6A	No	No
H13	Histone H1.3 GN = HIST1H1D	No	Iso
H2A1B	Histone H2A type 1-B/E GN = HIST1H2AB	No	Yes
H2B1M;H2B1A	Histone H2B type 1-M GN = HIST1H2BM	No	Yes
H4	Histone H4 GN = HIST1H4A	No	Yes
HBA	Hemoglobin subunit α GN = HBA1	No	Iso
HBB;HBD	Hemoglobin subunit β GN = HBB	No	Yes
HORN	Hornerin GN = HRNR	No	No
HS12A	Heat shock 70 kDa protein 12A GN = HSPA12A	No	Iso
HS71L	Heat shock 70 kDa protein 1-like GN = HSPA1L	No	Iso

HSPB1	Heat shock protein β1 GN = HSPB1	No	Iso
IGHG4;IGHG2	Ig γ-4 chain C region GN = IGHG4	No	Iso
IGHM	Ig μ chain C region GN = IGHM	No	Yes
IGKC	Ig κ chain C region GN = IGKC	No	Iso
KCC2A;KCC2D	Calcium/calmodulin-dependent protein kinase type II subunit α GN = CAMK2A	Yes	Yes
KCC2B;KCC2G	Calcium/calmodulin-dependent protein kinase type II subunit β GN = CAMKIIB	Yes	Yes
MBP	Myelin basic protein GN = MBP	Yes	Yes
ML12A;MYL9	Myosin regulatory light chain 12A GN = MYL12A	No	Iso
MOG	Myelin-oligodendrocyte glycoprotein GN = MOG	No	No
MPCP	Phosphate carrier protein, mito GN = SLC25A3	Yes	Yes
MYH10	Myosin-10 GN = MYH10	Yes	Yes
MYH9;MYH11;MYH14	Myosin-9 GN = MYH9	Iso	Yes
MYL6;MYL6B	Myosin light polypeptide 6 GN = MYL6	Yes	Yes
MYO5A;MYO5B	Unconventional myosin-Va GN = MYO5A	No	Yes
MYPR	Myelin proteolipid protein GN = PLP1	No	Yes
NDUA4	Cytochrome c oxidase subunit NDUFA4 GN = NDUFA4	Yes	Iso
NDUS3	NADH dehydrogenase [ubiquinone] iron-sulfur protein 3, mito GN = NDUFS3	Yes	Yes
NDUV2	NADH dehydrogenase [ubiquinone] flavoprotein 2, mito GN = NDUFV2	Yes	Yes
NFL	Neurofilament light polypeptide GN = NEFL	Yes	Yes
NFM	Neurofilament medium polypeptide GN = NEFM	Yes	Yes
NSF	Vesicle-fusing ATPase GN = NSF	No	Yes
ODB2	Lipoamide acyltransferase of branched-chain α-keto acid dehydrogenase mito GN = DBT	No	Yes
ODO2	Dihydrolipoyllysine-residue succinyltransferase 2-oxoglutarate dehydrogenase mito GN = DLST	Yes	Yes
QCR2	Cytochrome b-c1 complex subunit 2, mito GN = UQCRC2	No	Yes
RAB1A	Ras-related protein Rab-1A GN = RAB1A	Iso	Yes
RL40	Ubiquitin-60S ribosomal protein L40 GN = UBA52	No	No
RO52	E3 ubiquitin-protein ligase TRIM21 GN = TRIM21	No	No
S10A7;S1A7A	Protein S100-A7 GN = S100A7	No	No
S10A8	Protein S100-A8 GN = S100A8	No	No
S10A9	Protein S100-A9 GN = S100A9	No	No
SIR2	NAD-dependent protein deacetylase sirtuin-2 GN = SIRT2	No	No
SNP25	Synaptosomal-associated protein 25 GN = SNAP25	Yes	Yes
SPTB2;SPTB1	Spectrin β chain, nonerythrocytic 1 GN = SPTBN1	Yes	Yes
SPTN1	Spectrin α chain, nonerythrocytic 1 GN = SPTAN1	Yes	Yes
SPTN2	Spectrin β chain, nonerythrocytic 2 GN = SPTBN2	Iso	Iso
SSBP	Single-stranded DNA-binding protein, mito GN = SSBP1	No	No
STX1A	Syntaxin-1A GN = STX1A	Iso	Iso
STX1B;STX2	Syntaxin-1B GN = STX1B	Yes	Yes
STXB1	Syntaxin-binding protein 1 GN = STXBP1	No	Yes
SV2A	Synaptic vesicle glycoprotein 2A GN = SV2A	No	No
SYT1	Synaptotagmin-1 GN = SYT1	Yes	Yes
TBA1A,C;TBA3C,E;TBA8	Tubulin α1A chain GN = TUBA1A	Iso	Yes
TBA1B	Tubulin α1B chain GN = TUBA1B	Iso	Iso
TBA4A;TBA4B;TBAL3	Tubulin α4A chain GN = TUBA4A	Yes	Yes
TBB2A;TBB1;TBB2B	Tubulin β2A chain GN = TUBB2A	Yes	Yes
TBB3	Tubulin β3 chain GN = TUBB3	Yes	Yes
TBB4A;TBB6,8;TBB8L	Tubulin β4A chain GN = TUBB4A	Yes	Yes
TBB4B	Tubulin β4B chain GN = TUBB4B	Yes	Yes
TBB5	Tubulin β chain GN = TUBB	Yes	Yes
THY1	Thy-1 membrane glycoprotein GN = THY1	Yes	No
TMOD2	Tropomodulin-2 GN = TMOD2	No	Yes
TPM4;TPM1	Tropomyosin α4 chain GN = TPM4	Iso	No
VA0D1;VA0D2	V-type proton ATPase subunit d 1 GN = ATP6V0D1	Yes	Iso
VAMP3	Vesicle-associated membrane protein 3 GN = VAMP3	No	Iso
VATA	V-type proton ATPase catalytic subunit A GN = ATP6V1A	Yes	Yes
VISL1	Visinin-like protein 1 GN = VSNL1	No	No
VPP1	V-type proton ATPase 116 kDa subunit a isoform 1 GN = ATP6V0A1 PE = 2	Yes	Iso

**Table 2.** Statistical analyses indicating significance of variance with levels of the β2-nAChR subunit

Accession_HUMAN	ANOVA (*p*)	Peptides
ACTB;ACTBM;POTEE;POTEF;POTEI;POTEJ	1.10E-11	30
S10A9	1.67E-11	5
ODO2	2.50E-11	2
ACTC;ACTA	1.51E-10	1
BASP1	2.15E-10	2
ACTN2	1.03E-09	5
KCC2A;KCC2D	2.14E-09	11
TBA4A;TBA4B;TBAL3	2.40E-09	3
TBA1A;TBA1C;TBA3C;TBA3E;TBA8	2.92E-09	2
MYH9;MYH11;MYH14	3.28E-09	6
S10A7;S1A7A	3.97E-09	2
AT1A1;AT12A	6.35E-09	7
AT1B1	9.37E-09	3
TBA1B	6.14E-08	2
VAMP3	2.62E-07	1
TBB4A;TBB6;TBB8;TBB8L	3.00E-07	10
H2B1M;H2B1A	3.68E-07	4
RL40	8.67E-07	2
MYH10	1.11E-06	18
FCGBP	1.34E-06	19
ODB2	2.48E-06	5
COX41	2.57E-06	2
GELS	3.76E-06	10
HS12A	3.82E-06	4
AT1A3;AT1A4;ATP4A	4.09E-06	19
TBB2A;TBB1;TBB2B	4.12E-06	25
ACTN1;ACTN3	1.03E-05	19
TBB5	1.04E-05	2
ADT1;ADT2;ADT4	1.09E-05	3
IGHG4;IGHG2	1.34E-05	3
EF1A1;EF1A2	1.53E-05	2
DREB	2.67E-05	5
COX2	3.01E-05	2
CLH1	4.69E-05	2
CNTN1	5.48E-05	1
IGKC	5.53E-05	1
STX1B;STX2	6.30E-05	6
H13	8.79E-05	1
EAA2	1.11E-04	2
CX7A2	1.19E-04	1
ANK2	1.43E-04	3
VISL1	1.84E-04	2
MYO5A;MYO5B	2.45E-04	8
ML12A;MYL9	4.10E-04	4
S10A8	4.70E-04	2
ATPA	5.39E-04	3
ACTN4	5.80E-04	5
GFAP	6.13E-04	16
ARPC4	6.76E-04	2
ACHB2	7.24E-04	10
ARPC3	1.22E-03	1
CANB1	1.35E-03	1
STXB1	1.55E-03	3
TBB4B	1.58E-03	1
TBB3	1.73E-03	2
MPCP	2.04E-03	1
1433Z	2.60E-03	2
H4	2.92E-03	3
MYPR	2.96E-03	5
NDUS3	3.08E-03	1

GPM6A	3.13E-03	2
E41L3	3.53E-03	1
CALM	3.66E-03	2
H2A1B	4.17E-03	1
FRIH	4.43E-03	3
SPTN1	4.48E-03	57
GNAO;GNA12	4.51E-03	4
ACHA4;ACHA2;ACHA6	5.52E-03	23
DYN1	5.67E-03	4
ARP3	5.99E-03	2
SIR2	6.01E-03	1
HS71L	7.68E-03	1
ARP2	8.71E-03	4
SPTB2;SPTB1	0.013000	48
QCR2	0.019305	1
SPTN2	0.019379	3
AP1B1	0.023924	1
VA0D1;VA0D2	0.026761	4
1433E	0.028173	1
HBB;HBD	0.031713	4
AT2B2;AT2B1	0.033005	2
HORN	0.037459	2
VPP1	0.047673	2
MBP	0.053508	6
1433G;1433F	0.066781	2
TMOD2	0.066906	1
IGHM	0.067934	1
SV2A	0.069290	1
KCC2B;KCC2G	0.070110	2
ATPB	0.074512	3
RAB1A	0.076581	1
FRIL	0.083836	1
VATA	0.089288	1
ADDA	0.093382	2
AT1A2	0.126331	3
RO52	0.126544	12
EAA1	0.131182	1
1433S	0.149390	1
STX1A	0.155190	4
SNP25	0.193139	7
ALBU	0.249829	2
HBA	0.254867	2
SYT1	0.257254	2
G3P	0.263379	3
MYL6;MYL6B	0.365416	5
GBB1;GBB2;GBB3;GBB4	0.378267	7
TPM4;TPM1	0.409469	3
COX6C	0.456303	1
NDUA4	0.511653	2
CX6B1	0.580761	1
SSBP	0.640567	2
NSF	0.666753	8
MOG	0.744318	2
NFM	0.769224	2
THY1	0.778619	2
NDUV2	0.802323	1
NFL	0.842694	5
HSPB1	0.941399	1

**Table 3.** Proteins coimmunopurified with β2*-nAChRs from postmortem human temporal cortex with abundances that correlate significantly with the abundance of the β2 nAChR subunit

Accession	Description	Correlation (*r*)	*p* Value	*n*
ACHB2_HUMAN	Neuronal acetylcholine receptor subunit β2	1	0	18
ACHA4_HUMAN	Neuronal acetylcholine receptor subunit α4	0.972	<0.0009	18
AT1B1_HUMAN	Sodium/potassium-transporting ATPase subunit β1	0.889	<0.0009	18
AT1A3_HUMAN	Sodium/potassium-transporting ATPase subunit α3	0.888	<0.0009	18
SNP25_HUMAN	Synaptosomal-associated protein 25	0.88	<0.0009	18
STX1B_HUMAN	Syntaxin-1B	0.874	<0.0009	18
AT1A1_HUMAN	Sodium/potassium-transporting ATPase subunit α1	0.871	<0.0009	18
ANK2_HUMAN	Ankyrin-2	0.856	<0.0009	18
STX1A_HUMAN	Syntaxin-1A	0.844	<0.0009	18
GPM6A_HUMAN	Neuronal membrane glycoprotein M6-a	0.823	<0.0009	18
AT2B2_HUMAN	Plasma membrane calcium-transporting ATPase 2	0.781	<0.0009	18
GNAO_HUMAN	Guanine nucleotide-binding protein G(o) subunit α	0.781	<0.0009	18
VPP1_HUMAN	V-type proton ATPase 116 kDa subunit a isoform 1	0.777	<0.0009	18
MYPR_HUMAN	Myelin proteolipid protein	0.762	<0.0009	18
GBB1_HUMAN	Guanine nucleotide-binding protein G(I)/G(S)/G(T) subunit β1	0.702	0.001	18
EAA2_HUMAN	Excitatory amino acid transporter 2	0.701	0.001	18
VA0D1_HUMAN	V-type proton ATPase subunit d 1	0.644	0.004	18
MYH9_HUMAN	Myosin-9	0.631	0.005	18
AT1A2_HUMAN	Sodium/potassium-transporting ATPase subunit α2	0.609	0.007	18
TBB5_HUMAN	Tubulin β chain	0.576	0.012	18
TBA1A_HUMAN	Tubulin α1A chain	0.56	0.016	18
1433Z_HUMAN	14-3-3 protein zeta/delta	0.548	0.019	18
STXB1_HUMAN	Syntaxin-binding protein 1	0.526	0.025	18
SYT1_HUMAN	Synaptotagmin-1	0.523	0.026	18
CLH1_HUMAN	Clathrin heavy chain 1	0.515	0.041	18
TBA4A_HUMAN	Tubulin α4A chain	0.514	0.029	18
S10A7_HUMAN	Protein S100-A7	0.511	0.03	18
TBA1B_HUMAN	Tubulin α1B chain	0.506	0.032	18
RO52_HUMAN	E3 ubiquitin-protein ligase TRIM21	0.492	0.038	18
ODO2_HUMAN	Dihydrolipoyllysine-residue succinyltransferase component of 2-oxoglutarate dehydrogenase complex	0.483	0.042	18
TBB4A_HUMAN	Tubulin β4A chain	0.476	0.046	18
MYO5A_HUMAN	Unconventional myosin-Va	0.475	0.046	18
TPM4_HUMAN	Tropomyosin α4 chain	0.473	0.047	18

High correlation indicates the identified protein is either specifically interacting with the receptor at the time of tissue lysis or is part of a complex that stays intact following extraction in Triton X-100. The cluster of proteins with correlation coefficients <0.7 is evidence of significant enrichment of presynaptic components critical for neurotransmitter release and neuronal excitability.

### Immunoprecipitation of β2*-nAChRs from mouse cortex and identification of specific interacting proteins by tandem mass spectrometry

**Figure 3.**
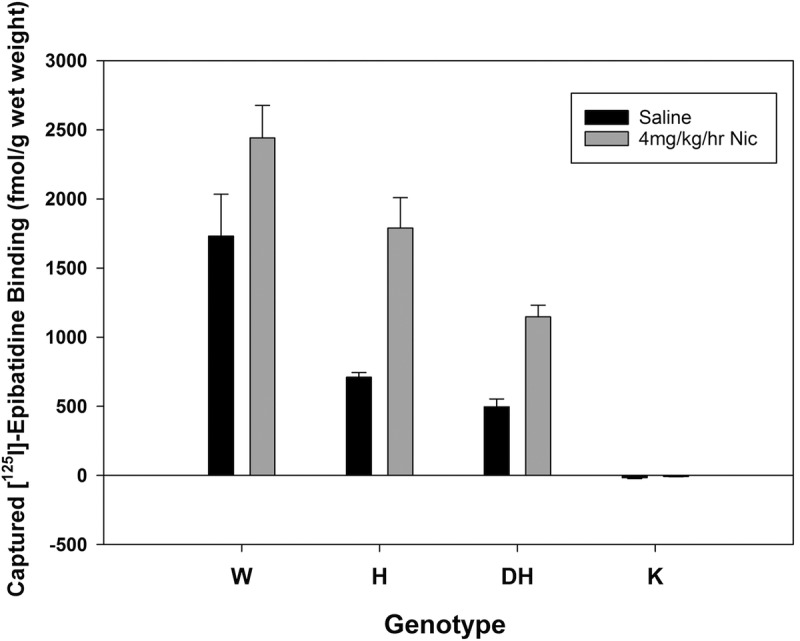
Effects of long-term treatment with saline or 4 mg/kg/h nicotine treatment on captured [^125^I]-epibatidine binding sites from pooled cortex from wild-type (β2^+/+^, W), heterozygous (β2^+/−^, H), double-heterozygous (α4^+/−^β2^+/−^, DH), and knock-out (β2^−/−^, K) mice. Gene dose-dependent changes in receptor capture are apparent (two-way ANOVA; main effect, genotype: *F*_(3,15)_ = 59.314, *p* < 0.0009), as is the upregulation produced by long-term nicotine exposure in all but the β2^−/−^ genotype groups (main effect, nicotine: *F*_(1,15)_ = 29.05, *p* < 0.0009). Error bars represent standard error of the mean.

As for the human samples, β2 nAChR binding sites in mouse cortex samples were immunoprecipitated by mAb295-Dynabeads and subsequently quantitated by [I^125^]-epibatidine binding. Binding site capture normalized to total tissue weight demonstrated significant effects of nicotine treatment and genotype (Fig. 3), but not a significant interaction (two-way ANOVA; main effect, nicotine, *F*_(1,15)_ = 29.05, *p* < 0.0009; main effect, genotype: *F*_(3,15)_ = 59.314, *p* < 0.0009; interaction, *F*_(3,15)_ = 3.846, *p* = 0.057).

Proteins identified and quantified from mouse cortex were analyzed, as has been described by McClure-Begley et al. (2013), using identical criteria for inclusion and exclusion as the human tissue samples. Each mouse sample was pooled from three mice (two pools per condition × eight conditions [nicotine-treated and untreated mice; wild-type, heterozygous, double-heterozygous, and knock-out mice]), and we mixed the mouse tissue such that each sample contained a total of ∼0.62 g wet weight. As expected, nAChR abundance calculated from the depletion of specific [^125^I]-epibatidine binding sites (as femtomoles/milligram protein) correlated very well with the iTRAQ abundance ratios of α4- and β2-nAChR subunits (*r* ^2^ = 0.77 and 0.70, respectively).

Identified proteins were considered to be significant interactors according to the overall correlation of the log2-transformed ratios of mean iTRAQ reporter ions with the control sample for each set (in these samples, the control is tissue from wild-type animals; Fig. 3, WT treated with saline). We observed a total of 184 coimmunopurified proteins from the mouse cortex samples, and, after the elimination of obvious contaminants and proteins identified in only one biological replicate, there were 94 proteins in common between the human and mouse experiments (Table 4). Of the 94 proteins identified in both biological replicates (Table 4), 5 were significantly correlated with β2-nAChR subunit abundance; statistical power was reduced compared with the human samples because individuals in the mouse study were pooled for measurement rather than used as biological replicates (Table 5). In addition, the comparative mouse nAChR-associated proteomes were collected using a peptide-labeling iTRAQ strategy, which has been shown previously to provide less comprehensive sequence coverage and fewer quantitative differences than LFQ (Latosinska et al., 2015). Of the 94 proteins identified in the nAChR interactome in mouse cortex, only 19 were not identified in the human cortex samples directly, or as a close isoform (Table 4). Further comparison to proteins associated with the β2-nAChR subunit in whole mouse brain (McClure-Begley et al., 2013) identified 37 proteins that were also immunoprecipitated from mouse and human cortex (Table 6). Comparing these proteins to those present in a common repository of contaminant proteins (Mellacheruvu et al., 2013), 11 proteins were identified <10 times in control studies, 7 were identified in <100 studies, 16 were identified in >100 control samples, and 3 were not present in the database (Table 6).

**Table 4.** Tabulation of all proteins identified in common between two biological replicates of eight-plex iTRAQ-labeled β2*-nAChRs immunoisolated with mAb295 from mouse cortex, indicating which proteins have been identified in human temporal cortex or whole mouse brain (Iso, isoform of protein identified; GN, gene name)

Accession_MOUSE	Description	Human Ctx	Mouse brain
1433T	14-3-3 protein theta GN = Ywhaq	Iso	Iso
ACHA4	Neuronal acetylcholine receptor subunit α4 GN = Chrna4	Yes	Yes
ACHA5	Neuronal acetylcholine receptor subunit α5 GN = Chrna5	Iso	Iso
ACHB2	Neuronal acetylcholine receptor subunit β2 GN = Chrnb2	Yes	Yes
ACPM	Acyl carrier protein, mito GN = Ndufab1	No	Yes
ACTG; ACTB	Actin, cytoplasmic 2 GN = Actg1; actin, cytoplasmic 1 GN = Actb	Iso	Yes
ADT1	ADP/ATP translocase 1 GN = Slc25a4 SV = 4	Yes	Yes
ADT2	ADP/ATP translocase 2 GN = Slc25a5	Yes	Yes
AINX	α-Internexin GN = Ina	No	Yes
AT1A3	Sodium/potassium-transporting ATPase subunit α3 GN = Atp1a3	Yes	Yes
AT1B1	Sodium/potassium-transporting ATPase subunit β1 GN = Atp1b1	Yes	Yes
AT5F1	ATP synthase subunit b, mito GN = Atp5f1	Iso	Yes
ATP5H	ATP synthase subunit d, mito GN = Atp5h	Iso	Yes
ATP5I	ATP synthase subunit e, mito GN = Atp5i	Iso	Yes
ATPA	ATP synthase subunit α, mito GN = Atp5a1	Yes	Yes
ATPB	ATP synthase subunit β, mito GN = Atp5b	Yes	Yes
ATPD	ATP synthase subunit delta, mito GN = Atp5d	Iso	Yes
C1QA	Complement C1q subcomponent subunit A GN = C1qa	No	No
CALM	Calmodulin GN = Calm1	Yes	Yes
CLH1	Clathrin heavy chain 1 GN = Cltc	Yes	No
CMC1	Calcium-binding mito carrier protein Aralar1 GN = Slc25a12	No	Yes
CN37	2',3'-cyclic-nucleotide 3'-phosphodiesterase GN = Cnp	No	Yes
EAA2	Excitatory amino acid transporter 2 GN = Slc1a2	Yes	Yes
G3P	Glyceraldehyde-3-phosphate dehydrogenase GN = Gapdh	Yes	Yes
GBB1	Guanine nucleotide-binding protein G(I)/G(S)/G(T) subunit β1 GN = Gnb1	Yes	Yes
GFAP	Glial fibrillary acidic protein GN = Gfap SV = 4	Yes	Yes
GLNA	Glutamine synthetase GN = Glul SV = 6	No	Yes
GNAO	Guanine nucleotide-binding protein G(o) subunit α GN = Gnao1	Yes	Yes
KCC2A	Calcium/calmodulin-dependent protein kinase type II subunit α GN = CaMKIIa	Yes	Yes
KCC2B	Calcium/calmodulin-dependent protein kinase type II subunit β GN = CaMKIIb	Yes	Yes
M2OM	Mito 2-oxoglutarate/malate carrier protein GN = Slc25a11	No	Yes
MBP	Myelin basic protein GN = Mbp	Yes	Yes
MPCP	Phosphate carrier protein, mito GN = Slc25a3	Yes	Yes
MYH10	Myosin-10 GN = Myh10	Yes	Yes
MYL6	Myosin light polypeptide 6 GN = Myl6	Yes	Yes
NDUA2	NADH dehydrogenase [ubiquinone] 1 α subcomplex subunit 2 GN = Ndufa2	Iso	Yes
NDUA4	NADH dehydrogenase [ubiquinone] 1 α subcomplex subunit 4 GN = Ndufa4	Yes	Iso
NDUA5	NADH dehydrogenase [ubiquinone] 1 α subcomplex subunit 5 GN = Ndufa5	Iso	Yes
NDUA6	NADH dehydrogenase [ubiquinone] 1 α subcomplex subunit 6 GN = Ndufa6	Iso	Yes
NDUA7	NADH dehydrogenase [ubiquinone] 1 α subcomplex subunit 7 GN = Ndufa7	Iso	Yes
NDUA9	NADH dehydrogenase [ubiquinone] 1 α subcomplex subunit 9, mito GN = Ndufa9	Iso	Yes
NDUAA	NADH dehydrogenase [ubiquinone] 1 α subcomplex subunit 10, mito GN = Ndufa10	Iso	Yes
NDUAC	NADH dehydrogenase [ubiquinone] 1 α subcomplex subunit 12 GN = Ndufa12	Iso	Yes
NDUAD	NADH dehydrogenase [ubiquinone] 1 α subcomplex subunit 13 GN = Ndufa13	Iso	Yes
NDUB3	NADH dehydrogenase [ubiquinone] 1 β subcomplex subunit 3 GN = Ndufb3	Iso	Yes
NDUB4	NADH dehydrogenase [ubiquinone] 1 β subcomplex subunit 4 GN = Ndufb4	Iso	Yes
NDUB5	NADH dehydrogenase [ubiquinone] 1 β subcomplex subunit 5, mito GN = Ndufb5	Iso	Yes
NDUB6	NADH dehydrogenase [ubiquinone] 1 β subcomplex subunit 6 GN = Ndufb6	Iso	Yes
NDUB7	NADH dehydrogenase [ubiquinone] 1 β subcomplex subunit 7 GN = Ndufb7	Iso	Yes
NDUB8	NADH dehydrogenase [ubiquinone] 1 β subcomplex subunit 8, mito GN = Ndufb8	Iso	Yes
NDUB9	NADH dehydrogenase [ubiquinone] 1 β subcomplex subunit 9 GN = Ndufb9	Iso	Yes
NDUC2	NADH dehydrogenase [ubiquinone] 1 subunit C2 GN = Ndufc2	Iso	Yes
NDUS1	NADH-ubiquinone oxidoreductase 75 kDa subunit, mito GN = Ndufs1	Iso	Yes
NDUS2	NADH dehydrogenase [ubiquinone] iron-sulfur protein 2, mito GN = Ndufs2	Iso	Yes
NDUS3	NADH dehydrogenase [ubiquinone] iron-sulfur protein 3, mito GN = Ndufs3	Yes	Yes
NDUS4	NADH dehydrogenase [ubiquinone] iron-sulfur protein 4, mito GN = Ndufs4	Iso	Yes
NDUS5	NADH dehydrogenase [ubiquinone] iron-sulfur protein 5 GN = Ndufs5	Iso	Yes
NDUS6	NADH dehydrogenase [ubiquinone] iron-sulfur protein 6, mito GN = Ndufs6	Iso	Yes

NDUS7	NADH dehydrogenase [ubiquinone] iron-sulfur protein 7, mito GN = Ndufs7	Iso	Yes
NDUS8	NADH dehydrogenase [ubiquinone] iron-sulfur protein 8, mito GN = Ndufs8	Iso	Yes
NDUV1	NADH dehydrogenase [ubiquinone] flavoprotein 1, mito GN = Ndufv1	Iso	Yes
NDUV2	NADH dehydrogenase [ubiquinone] flavoprotein 2, mito GN = Ndufv2	Yes	Yes
NEST	Nestin GN = Nes	No	No
NFH	Neurofilament heavy polypeptide GN = Nefh	Iso	Iso
NFL	Neurofilament light polypeptide GN = Nefl SV = 5	Yes	Yes
NFM	Neurofilament medium polypeptide GN = Nefm SV = 4	Yes	Yes
OCAD2	OCIA domain-containing protein 2 GN = Ociad2	No	Yes
ODO2	Dihydrolipoyl-lysine residue succinyltransferas 2-oxoglutarate dehydrogenase complex, mito GN = Dlst	Yes	Yes
ODPA	Pyruvate dehydrogenase E1 component subunit α, somatic form, mito GN = Pdha1	No	Yes
ODPB	Pyruvate dehydrogenase E1 component subunit β, mito GN = Pdhb	No	Yes
PHB	Prohibitin GN = Phb	No	Yes
PHB2	Prohibitin-2 GN = Phb2	No	Yes
RAB3A	Ras-related protein Rab-3A GN = Rab3a	Iso	Yes
SFXN3	Sideroflexin-3 GN = Sfxn3	No	Yes
SNP25	Synaptosomal-associated protein 25 GN = Snap25	Yes	Yes
SPTB2	Spectrin β chain, nonerythrocytic 1 GN = Sptbn1	Yes	Yes
SPTN1	Spectrin α chain, nonerythrocytic 1 GN = Sptan1 SV = 4	Yes	Yes
STX1B	Syntaxin-1B GN = Stx1b	Yes	Yes
SYPH	Synaptophysin GN = Syp	No	No
SYT1	Synaptotagmin-1 GN = Syt1	Yes	Yes
TBA4A	Tubulin α4A chain GN = Tuba4a	Yes	Yes
TBB2A	Tubulin β2A chain GN = Tubb2a	Yes	Yes
TBB3	Tubulin β3 chain GN = Tubb3	Yes	Yes
TBB4A	Tubulin β4A chain GN = Tubb4a	Yes	Yes
TBB4B	Tubulin β4B chain GN = Tubb4b	Yes	Yes
TBB5	Tubulin β5 chain GN = Tubb5	Yes	Yes
THY1	Thy-1 membrane glycoprotein GN = Thy1	Yes	No
TPM3	Tropomyosin α3 chain GN = Tpm3	Iso	No
VA0D1	V-type proton ATPase subunit d 1 GN = Atp6v0d1	Yes	Iso
VATA	V-type proton ATPase catalytic subunit A GN = Atp6v1a	Yes	Yes
VDAC1	Voltage-dependent anion-selective channel protein 1 GN = Vdac1	No	Yes
VDAC2	Voltage-dependent anion-selective channel protein 2 GN = Vdac2	No	Yes
VIME	Vimentin GN = Vim	No	Yes
VPP1	V-type proton ATPase 116 kDa subunit a isoform 1 GN = Atp6v0a1	Yes	Iso

**Table 5.** β2*-nAChR interacting proteins identified by iTRAQ analysis of receptors isolated from transgenic mouse cortex receiving long-term treatment with nicotine and not receiving nicotine treatment (GN, gene name)

Accession	Description	Correlation (*r*)	*p* Value
sp|O70174|ACHA4_MOUSE	Neuronal acetylcholine receptor subunit α4 OS = Mus musculus GN = Chrna4 PE = 2 SV = 2	0.97	<0.0009
sp|Q2MKA5|ACHA5_MOUSE	Neuronal acetylcholine receptor subunit α5 OS = Mus musculus GN = Chrna5 PE = 2 SV = 1	0.91	<0.0009
sp|Q9ERK7|ACHB2_MOUSE	Neuronal acetylcholine receptor subunit β2 OS = Mus musculus GN = Chrnb2 PE = 2 SV = 1	1	<0.0009
sp|Q6PIC6|AT1A3_MOUSE	Sodium/potassium-transporting ATPase subunit α3 OS = Mus musculus GN = Atp1a3 PE = 1 SV = 1	0.55	0.042
sp|Q62277|SYPH_MOUSE	Synaptophysin OS = Mus musculus GN = Syp PE = 1 SV = 2	0.59	0.026

Similar to the results from human cortex samples, presynaptic elements related to vesicle fusion (synaptophysin) and neuronal excitability (ATP1A3) are significantly correlated with nAChR levels.

**Table 6.** List of proteins identified in three independent proteomic studies: human and mouse cortex (current study) and whole mouse brain (McClure-Begley et al., 2013) (GN, gene name)

Accession	Description	No. of experiments (found/total)
ACHA4	Neuronal acetylcholine receptor subunit α4 GN = Chrna4	0/411
ACHB2	Neuronal acetylcholine receptor subunit β2 GN = Chrnb2	0/411
ADT1	ADP/ATP translocase 1 GN = Slc25a4	195/411
ADT2	ADP/ATP translocase 2 GN = Slc25a5	223/411
AT1A3	Sodium/potassium-transporting ATPase subunit α3 GN = Atp1a3	101/411
AT1B1	Sodium/potassium-transporting ATPase subunit β1 GN = Atp1b1	1/411
ATPA	ATP synthase subunit α, mito GN = Atp5a1	210/411
ATPB	ATP synthase subunit β, mito GN = Atp5b	211/411
CALM	Calmodulin GN = Calm1	133/411
EAA2	Excitatory amino acid transporter 2 GN = Slc1a2	2/411
G3P	Glyceraldehyde-3-phosphate dehydrogenase GN = GAPDH	248/411
GBB1	Guanine nucleotide-binding protein G(I)/G(S)/G(T) subunit β1 GN = Gnb1	59/411
GFAP	Glial fibrillary acidic protein GN = GFAP	73/411
GNAO	Guanine nucleotide-binding protein G(o) subunit α GN = Gnao1	42/411
KCC2A	Calcium/calmodulin-dependent protein kinase type II subunit α GN = CaMKIIa	7/411
KCC2B	Calcium/calmodulin-dependent protein kinase type II subunit β GN = CaMKIIb	7/411
MBP	Myelin basic protein GN = Mbp	0/411
MPCP	Phosphate carrier protein, mito GN = Slc25a3	130/411
MYH10	Myosin-10 GN = Myh10	166/411
MYL6	Myosin light polypeptide 6 GN = Myl6	190/411
NDUS3	NADH dehydrogenase [ubiquinone] iron-sulfur protein 3, mito GN = Ndufs3	19/411
NDUV2	NADH dehydrogenase [ubiquinone] flavoprotein 2, mito GN = Ndufv2	3/411
NFL	Neurofilament light polypeptide GN = Nefl	Not in database
NFM	Neurofilament medium polypeptide GN = Nefm	85/411
ODO2	Dihydrolipoyllysine-res succinyltransferase 2-oxoglutarate dehydrogenase complex, mito GN = Dlst	57/411
SNP25	Synaptosomal-associated protein 25 GN = Snap25	0/411
SPTB2	Spectrin β chain, nonerythrocytic 1 GN = Sptbn1	143/411
SPTN1	Spectrin α chain, nonerythrocytic 1 GN = Sptan1	161/411
STX1B	Syntaxin-1B GN = Stx1b	0/411
SYT1	Synaptotagmin-1 GN = Syt1	0/411
TBA4A	Tubulin α4A chain GN = Tuba4a	377/411
TBB2A	Tubulin β2A chain GN = Tubb2a	372/411
TBB3	Tubulin β3 chain GN = Tubb3	Not in database
TBB4A	Tubulin β4A chain GN = Tubb4a	366/411
TBB4B	Tubulin β4B chain GN = Tubb4b	376/411
TBB5	Tubulin β5 chain GN = Tubb5	Not in database
VATA	V-type proton ATPase catalytic subunit A GN = Atp6v1a	62/411

Numbers in the right hand column represent the number of times each protein has been identified in control studies designed to evaluate background contaminants (Mellacheruvu et al., 2013).

### Effects of nicotine exposure and mood disorder on nAChR-associated proteins isolated from human temporal cortex

**Figure 4.**
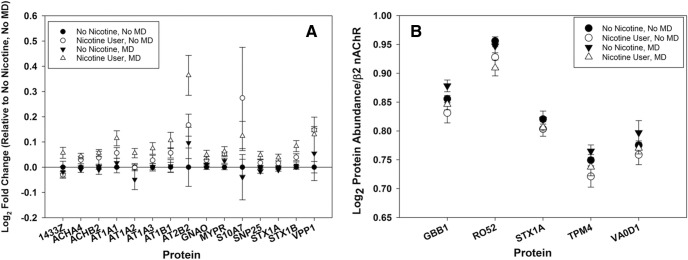
***A***, Representation of the log2-normalized fold change in β2*-nAChR-associated proteins in human temporal cortex relative to the non-nicotine, non-MD group average. The graph shows nAChR-associated proteins whose expression is significantly impacted by nicotine, MD, or an interaction, according to GLM. Group means above zero are upregulated by nicotine exposure, indicating that as nAChR levels increase, so do the levels of those proteins. Increases in excess of the log2 fold change for the α4- and β2-nAChR subunits indicate a superstoichiometric shift. ***B***, Log2 protein abundance normalized to within-sample β2-nAChR protein content for five proteins that showed a significant shift with nicotine use. Normalization demonstrates the change in protein expression per molecule of the nAChR β2-subunit in human cortical samples. For these samples, LFQ proteomics allows the comparison of protein levels, even when the variation in total abundance across treatments is small. Normalization to an internal standard in each condition (β2-subunit levels) allows the measurement of more discrete changes than are apparent from fold change estimates.

To identify changes in the β2*-nAChR-associated proteome due to nicotine exposure status or mood disorder, we compared the abundance ratios for every protein identified in each sample and performed GLM analysis to identify significant changes. Of the 33 putative interacting proteins identified following immunoprecipitation of β2*-nAChRs from human cortex (Table 3), 14 proteins differed in interaction as a result of tobacco use, as measured by a shift in the ratio of the interacting protein to nAChR number between nonexposed and tobacco-exposed individuals, with two additional protein (14-3-3ζ and ATP1A2) that showed a significant nicotine × mood disorder interaction (Fig. 4*A*, Table 7). None of the interactions were significantly altered by mood disorder alone (Table 7). Several of the changes in associated protein abundance appear to be affected more significantly by the combination of nicotine use and mood disorder (Fig. 4, open triangles) despite the nonsignificant interaction term calculated from the dataset as a whole. Normalizing protein abundance to β2 nAChR content within each sample identified an independent set of five proteins that were significantly altered by nicotine use (Fig. 4*B*, Table 8); again, we did not identify significant differences by mood disorder diagnosis.

**Table 7.** β2*-nAChR-associated proteins with abundances that are significantly affected by nicotine use and/or mood disorder status in human temporal cortex

Accession	Description	Significant effect	*F*	*p* Value
1433Z_HUMAN	14-3-3 protein zeta/delta	MD x Nicotine	*F*_(1,17)_ = 4.943	0.043
ACHA4_HUMAN	Neuronal acetylcholine receptor subunit α4	Nicotine	*F*_(1,17)_ = 16.596	<0.0009
ACHB2_HUMAN	Neuronal acetylcholine receptor subunit β2	Nicotine	*F*_(1,17)_ = 12.881	<0.0009
AT1A1_HUMAN	Sodium/potassium-transporting ATPase subunit α1	Nicotine	*F*_(1,17)_ = 7.460	0.016
AT1A2_HUMAN	Sodium/potassium-transporting ATPase subunit α2	Nicotine, MD × nicotine	*F*_(1,17)_ = 5.749; *F*_(1,17)_ = 4.740	0.031; 0.047
AT1A3_HUMAN	Sodium/potassium-transporting ATPase subunit α3	Nicotine	*F*_(1,17)_ = 5.820	0.03
AT1B1_HUMAN	Sodium/potassium-transporting ATPase subunit β1	Nicotine	*F*_(1,17)_ = 7.537	0.016
AT2B2_HUMAN	Plasma membrane calcium-transporting ATPase 2	Nicotine	*F*_(1,17)_ = 13.246	0.003
GNAO_HUMAN	Guanine nucleotide-binding protein G(o) subunit α	Nicotine	*F*_(1,17)_ = 5.077	0.041
MYPR_HUMAN	Myelin proteolipid protein	Nicotine	*F*_(1,17)_ = 8.779	0.01
S10A7_HUMAN	Protein S100-A7	Nicotine	*F*_(1,17)_ = 5.850	0.03
SNP25_HUMAN	Synaptosomal-associated protein 25	Nicotine	*F*_(1,17)_ = 11.990	0.004
STX1A_HUMAN	Syntaxin-1A	Nicotine	*F*_(1,17)_ = 11.506	0.004
STX1B_HUMAN	Syntaxin-1B	Nicotine	*F*_(1,17)_ = 11.792	0.004
VPP1_HUMAN	V-type proton ATPase 116 kDa subunit a isoform 1	Nicotine	*F*_(1,17)_ = 5.946	0.029

Nicotine use has the largest statistical effect observed in terms of its impact on β2*-nAChR expression and the abundance of associated proteins. Only two proteins (14-3-3ζ and ATP1A2) show a significant interaction between nicotine use and mood disorder.

**Table 8.** β2*-nAChR-associated proteins that show a significant effect of nicotine use on their “per-β2” abundance from human temporal cortex

Accession	Description	Significant effect	*F*	*p* Value
GBB1_HUMAN	Guanine nucleotide-binding protein G(I)/G(S)/G(T) subunit β1	Nicotine	*F*_(1,17)_ = 10.085	0.007
RO52_HUMAN	E3 ubiquitin-protein ligase TRIM21	Nicotine	*F*_(1,17)_ = 5.676	0.031
STX1A_HUMAN	Syntaxin-1A	Nicotine	*F*_(1,17)_ = 5.375	0.036
TPM4_HUMAN	Tropomyosin α4-chain	Nicotine	*F*_(1,17)_ = 5.911	0.029
VAOD1_HUMAN	V-type proton ATPase subunit d 1	Nicotine	*F*_(1,17)_ = 6.700	0.021

These proteins have abundance indices that correlate significantly with levels of β2-nAChR, but the effect of nicotine is apparent only when protein content is normalized to total β2-nAChR levels, suggesting that their expression does not track linearly with increases in nAChR due to nicotine-induced upregulation and likely represent limiting interactions.

### Effects of nicotine exposure on nAChR-associated proteins isolated from mouse cortex

In the mouse iTRAQ data, GLM analysis did not identify a significant effect of nicotine on any of the nAChR-associated proteins with expression indices significantly correlated with β2-nAChR abundance. Although nicotine exposure did increase the abundance of the α4- and β2-nAChR subunits, the increase was only marginally significant (Chrna4: *F*_(1,13)_ = 4.93, *p* = 0.06; Chrnb2: *F*_(1,13)_ = 4.67, *p* = 0.07), likely due to the small number of pools included in the analysis. In addition, the overall *n* size of the experiment was reduced because the β2-nAChR subunit abundance from the control groups is used as the denominator in their ratiometric quantitation, and is not included as a correlate to avoid bias due to artificially small errors on the calculated intercept. This ratiometric quantitation also prevents normalization to β2-nAChR subunit abundance within each sample, so a depiction of change relative to “per unit” β2 is not possible. Analysis of genotype effects on iTRAQ-labeled nAChR peptides with GLM showed significance for all three subunits identified, as follows: Chrna4: *F*_(3,13)_ = 16.43, *p* = 0.001; Chrna5: *F*_(3,13)_ = 8.87, *p* = 0.009; Chrnb2: *F*_(3,13)_ = 12.78, *p* = 0.003.

## Discussion

In this study, we examined the effects of tobacco use and mood disorder on the expression of α4β2*-nAChRs and their associated proteins in postmortem human cortex. We also used experiments in mice to determine the effects of nicotine alone on the associated proteome. Repeated or prolonged nicotine exposure increases α4β2*-nAChR number in heterologous expression systems, animal models, and human tissue (for review, see Melroy-Greif et al., 2016). In addition, the changes in α4β2*-nAChR content after long-term exposure to nicotine are brain region and cell type specific, with cortical regions among those most reproducibly affected in both mouse and human (Marks et al., 1992, 2011; Nashmi et al., 2007). Further, a substantial body of evidence points to a cholinergic contribution to the etiology and expression of affective disorders (for review, see Mineur and Picciotto, 2010), and that β2*-nAChR occupancy by endogenous ACh is significantly increased by current or past episodes of major depressive or bipolar disorder (Saricicek et al., 2012; Hannestad et al., 2013). Studies in mouse models indicate that even acute dysregulation of cholinergic neurotransmission can elicit changes in affective states, and that long-term manipulation of acetylcholine levels leads to behavioral responses that are commonly indicative of mood disorders, specifically depression- and anxiety-like behaviors (Mineur et al., 2013). Identifying the interactions of α4β2*-nAChRs with other synaptic proteins and functional regulators could therefore identify the novel elements of neuronal plasticity underlying the acquisition of nicotine tolerance and dependence, as well as improve understanding of how cholinergic regulation of mood is accomplished in the CNS.

For this study we measured cotinine levels in the brain tissue lysate (containing all of the cytosolic and interstitial fluid components) to establish nicotine/tobacco use status for each sample. After organizing the samples according to nicotine use and mood disorder status, we observed that the levels of α4β2*-nAChRs measured by radioligand binding with [^125^I]-epibatidine were consistent with significant receptor upregulation due to nicotine use. The levels of α4- and β2-nAChR subunits measured by label-free quantitative proteomics were consistent with the changes observed with ligand binding. These results indicated that, when performed appropriately, quantitative immunopurification and proteomics is a valid method for evaluating changes in nAChR expression.

A different form of quantitative proteomics (iTRAQ) was used to assess changes in α4β2*-nAChRs and associated proteins from transgenic mouse tissue. iTRAQ uses amine-reactive isobaric labels to facilitate the ratiometric quantitation of peptides from multiplexed samples in a complex mixture (Wiese et al., 2007). Multiplexed iTRAQ proteomics has been used previously with nAChR subunit transgenic mice to validate the approach for the untargeted identification of putative nAChR-associated proteins (McClure-Begley et al., 2013). In this project, we used iTRAQ to identify interacting proteins that are altered in their association with β2*-nAChR due to long-term nicotine treatment. It was our goal to test the hypothesis that a core set of α4β2* interacting partners would be conserved in the cortex from mouse to human, and that the identification of these complexes could guide future studies geared toward understanding nAChR regulation and function.

We took advantage of the inherent variability in β2*-nAChR abundance across samples (due to either individual differences and/or nicotine use status in the case of the human samples, and genetic control of nAChR abundance and nicotine exposure in the mouse samples) to match linear relationships of protein abundance indices with the measured abundance of β2-nAChRs, providing confidence that the proteins coimmunopurifying with the β2*-nAChRs interact in a meaningful context. Abundance correlations would be expected to be random and nonsignificantly correlated if interactions were nonspecific. We observed a high level of overlap between human and mouse cortical samples of signaling complexes coimmunoprecipitated with β2*-nAChRs.

The functional roles of nAChRs as neuromodulators on presynaptic terminals of many different neuronal types have been described in numerous previous studies (for review, see Barik and Wonnacott, 2009). Evidence in the current study from bottom-up quantitative proteomic analysis of α4β2*-nAChR-associated proteins identified in human and mouse cortical tissue not only supports a presynaptic role for these receptors, but assigns additional proteins with known roles in neurotransmitter release and neuronal excitability that begin to describe the composition of specific signaling complexes assembled with nAChRs. Specifically, components of the presynaptic active zone and the SNARE complex (syntaxin1B, syntaxin1A, syntaxin binding protein-1, SNAP25, and synaptotagmin) were identified with significant expression correlations with β2-nAChR subunits in the human temporal cortex samples; the same core set of proteins were also identified in the mouse cortex iTRAQ analysis. Smaller sample size due to the pooling of individual mice for analyses limited statistically significant correlation to synaptophysin; however, identification across two species with differing exposure modalities increases confidence that interaction of the set of nAChR-associated proteins identified from human brain are altered as a result of nicotine exposure.

Of the 17 proteins that were identified previously with iTRAQ using stringent criteria for dose relationships in mice with varying nAChR genotype in whole mouse brain (McClure-Begley et al., 2013), 8 were identified in both mouse and human cortex in the current study (α4- and β2-nAChR subunits, CaMKIIα, spectrin α and β, myosin 10, tubulin β3, and GFAP; Tables 1, 2). Syntaxin 1B, synaptotagmin 1, syntaxin binding protein 1, and several 14-3-3 isoforms were also identified in all three datasets. These proteins, although clearly identified in the nAChR-associated proteome from whole mouse brain, did not meet the stringent criteria for variance with gene dose. One possibility is that each of these proteins is limiting in the compartment (synaptic or ER) in which it interacts with nAChRs, and that the interaction is functional and transient (during vesicle recruitment or release, or during nAChR assembly), so it does not scale with nAChR number. Of those proteins that were not identified in the current study, several proteins were represented by different isoforms or by other proteins with related functions. For example, while the current study did not identify CaMKIIγ, the β-isoform was identified in both mouse and human cortical samples, perhaps because the γ-isoform is more highly expressed in other brain regions, and the β-isoform in enriched in cortex.

Many subtypes of nAChRs have been identified as presynaptic heteroreceptors that can directly influence neurotransmitter release, and the identification of SNARE complex proteins as significant nAChR interactors in both the mouse and human tissue samples suggests a functional interaction between α4β2*-nAChRs and neurotransmitter release machinery. Minimally, this association indicates that some α4β2*-nAChRs are extremely close to sites on presynaptic terminals where neurotransmitter release is actively taking place. Such close physical association suggests that nAChRs may exert some modulatory influence on neurotransmitter release independently of action potential arrival or frequency, and highlights their potential role as a “fine-tuning” influence on activity at highly specialized synapses. It is also possible that the significant interaction of synaptic vesicle components with β2*-nAChRs indicates the presence of α4β2*-nAChR-mediated neurotransmitter release process from perisynaptic sites, similar to what has been proposed for α7 and α3* nAChRs in the chick ciliary ganglion (Coggan et al., 2005). Interestingly, the degree of interaction between syntaxin1A and β2*-nAChRs was reduced in human cortical tissue from nicotine users, raising the possibility that long-term nicotine use promotes a functional reorganization of presynaptic nAChR signaling complexes. The family of 14-3-3 isoforms have multiple functions (for review, see Yaffe, 2002), including acting as chaperones and adaptors that mediate complex protein–protein interactions, and have been shown to facilitate nAChR trafficking and assembly of α3* nAChRs at postsynaptic sites in the ciliary ganglion (Rosenberg et al., 2008). The current finding showing that 14-3-3ζ interacts with β2*-nAChRs suggests that adapter protein interactions are critical determinants of trafficking and functional regulation of nAChRs on both presynaptic and postsynaptic plasma membranes and that these interactions are likely to be context specific. Nicotine use may also induce restructuring of postsynaptic elements of cholinergic synapses, but in this study we did not reliably identify components of larger postsynaptic complexes associated with β2*-nAChRs. It is possible that in the cortex of both mice and humans, the postsynaptic β2*-nAChR interactome is more dynamic and less likely to survive the conditions used for isolating nAChRs in this study; additional studies using methods for enriching and isolating nAChRs from specific subcellular neuronal compartments is necessary to address this question.

The proteoglycan agrin, which contributes to the orchestration of nAChR clustering at the neuromuscular junction (NMJ; Wallace et al., 1991), has also been identified as an inhibitor of α3-containing Na/K ATPases (Hilgenberg et al., 2006). We identified α3 Na/K-ATPase as a β2*-nAChR-interacting protein from both human and mouse cortical tissues. Its abundance was upregulated along with the β2*-nAChR subunit in the human samples with nicotine treatment, as evidenced by the similar ratio of α3 Na/K-ATPase/β2-nAChR levels across treatment groups. This parallel increase suggests that aspects of presynaptic neuronal excitability are changed by long-term nicotine treatment beyond the alterations in excitation due to prolonged nAChR desensitization or other mechanisms of agonist-induced functional changes. An additional link between altered Na/K-ATPase activity and mood disorder is found in the Myshkin mouse model, which harbors a loss-of-function mutation in the α3Na/K-ATPase and exhibits behavioral hallmarks of the manic phase of bipolar disorder that are responsive to traditional pharmacotherapies (valproate, lithium) as well as inhibitors of downstream signaling linked to α3Na/K-ATPase (Kirshenbaum et al., 2011). Postsynaptic nAChRs at the NMJ copurify with the α2Na/K-ATPase subunit (Heiny et al., 2010; Talsma et al., 2014), and we observe significant correlation here of α2-Na/K ATPase abundance with β2*-nAChRs. Genetic risk variants for mood disorders have been found in the Na/K ATPase α2- and α3-loci (Talsma et al., 2014), highlighting the potential functional interactions between nAChRs and Na/K-ATPases in the CNS that may also impact lifetime risk for developing mood disorders. The fact that we observe α4β2*-nAChRs associated with Na/K-ATPase subunits as well as SNARE complex machinery and other mediators of synaptic activity leads us to consider that presynaptic nAChRs may exist as part of a core activity complex with substantial homology to what has been shown to exist postsynaptically at the NMJ (Heiny et al., 2010; Talsma et al., 2014). Further experiments and fine-detailed structural analysis of presynaptic nAChR scaffolds in the CNS will, it is hoped, add to this concept of conserved cholinergic regulation via novel functions of proteins previously unknown as functional regulators of nAChRs.

Another specific β2-interactor of interest for additional study is the protein TRIM21, an E3 ubiquitin ligase with roles in cell cycle progression and immune signaling (Wada and Kamitani, 2006). Muscle-type nAChR turnover is at least partially dependent on the targeted autophagy of ubiquitinated receptors (Khan et al., 2014), and the role of the ubiquitin–proteasome system in the trafficking and stability of α3* nAChRs has been shown in both cell lines (Rezvani et al., 2010) and rodent neurons (Teng et al., 2015). Different E3 ubiquitin ligases were determined to be critical mediators of the selective degradation of ubiquitinylated muscle and α3* nAChR subtypes in the previous studies (TRIM63 and CHIP, respectively); our identification of TRIM21 as a β2*-nAChR-associated E3 ubiquitin ligase in human cortical neurons indicates that future studies are warranted to determine whether it is a specific regulator of α4β2*-nAChR turnover.

A recent publication presents a hypothesis that nAChRs, including β2*-nAChR, may possess a structural motif that promotes functional interaction with Gα subunits of the heterotrimeric G-protein complex (Kabbani et al., 2013). Earlier studies using proteomic methods to interrogate β2-nAChR subunit interactions identified Gα subunits as a reliable associated protein in mouse brain tissue lysate (Kabbani et al., 2007). Here we show that β2*-nAChRs from human cortical tissue also associate with Gα subunits (particularly, the Gα_o_ subtype), and that nicotine use significantly affects their abundance index across sample groups. Whether this change in Gα_o_ interaction with β2*-nAChRs is functionally relevant to the physiological response to short-term or long-term nicotine use remains to be elucidated, but our results supporting the earlier discovery that a β2–Gα interaction is taking place provides considerable support for additional studies aimed at dissecting the roles of metabotropic signaling via nAChR ion channels.

Of the identified proteins, only two, 14-3-3ζ and ATP1A2, showed a significant nicotine × mood disorder interaction. As noted above, the Na/K ATPase is critically involved in neuronal energetics and at the presynaptic terminal and variants in the ATPase have been associated with mood disorders (Talsma et al., 2014). In contrast, 14-3-3 proteins are involved in chaperoning nAChRs from the endoplasmic reticulum to the cell surface, and this chaperone function is regulated by protein phosphorylation (Jeanclos et al., 2001). The significant nicotine × mood disorder interaction in the association of these proteins with β2*-nAChRs may therefore suggest that nAChR trafficking is altered differentially by nicotine exposure in individuals with mood disorders. This is an intriguing hypothesis that can be evaluated in future studies.

Our data have identified several proteins involved in the regulation of neuronal excitation and neurotransmitter exocytosis that are proximally associated with nAChRs in humans and mice, and are significantly affected by physiological conditions known to impact the expression of nAChRs themselves. While mood disorders have been demonstrated to involve significant contributions from cholinergic components, the impacts of smoking status on nAChR expression as well as the expression of interacting proteins appears to be a much more powerful perturbation. It is possible that different mood disorders (i.e., bipolar disorder vs major depression vs general anxiety disorder) are also representative of distinct entities with respect to cholinergic dysfunction that would benefit from additional exploration of discrete changes in the nAChR-associated proteome. These results indicate that the regulation of nAChRs and other membrane signaling complexes in skeletal muscle share core regulatory and functional interactions with β2*-neuronal nAChRs, albeit with different proteins that serve functionally similar roles. This study shows that proteomic methods are a powerful tool to identify proteins associated with nAChRs. Proteomic strategies were different for the human and mouse β2-nAChR-interacting protein identifications, and, despite obvious technical distinctions, strengths, and weaknesses, remarkably conserved protein complexes were found to be associated with assembled receptors. Additional proteomic studies aimed at a finer dissection of cell type-specific nAChR interactions may identify more targets that regulate and are regulated by nAChR activity to provide additional mechanistic insight into the contribution of cholinergic dysfunction in neurological disorders.

## Synthesis

The decision was a result of the Reviewing Editor Alfonso Araque and the peer reviewers coming together and discussing their recommendations until a consensus was reached. A fact-based synthesis statement explaining their decision and outlining what is needed to prepare a revision is listed below. The following reviewers agreed to reveal their identity: Michele Jacob, Darwin Berg

The reviewers have found merit and interest on the findings reported. I concur with the reviewers' comments expressing the interest, novelty and quality of the study. However, they also expressed some concerns about several issues that need to be addressed and clarified. I also consider pertinent the concerns expressed. I believe that addressing those concerns, which will definitely improve the manuscript, is necessarily required to properly support the conclusions.

Specific comments of the reviewers:

Reviewer 1.

This manuscript addresses the important question of how do exposure to tobacco/nicotine and depressive mood disorders alter the levels of b2*nAChRs and their associated protein interactome in the human brain. They use an unbiased proteomic screen of postmortem human temporal cortex. For comparison to human b2 interactors, the study makes clever use of b2*nAChR mutant mice, heterozygous and homozygous deletions versus wild-type b2, plus versus minus nicotine exposure. The findings identify a relatively small number of proteins that both co-precipitate with b2 and show significantly altered levels in tobacco users versus non-users. Only two putative interactors, 14-3-3zeta and ATP1A2 differed significantly with both tobacco use and mood disorders. This finding extends reports that nicotine exposure alters other Na/K-ATPase isoforms in skeletal muscle. The current findings also extend reports that selected nAChR subunits link with particular isoforms of two other putative interactors (TRIM21, an E3 ubiquitin ligase and a Galpha subtype). However, the current study does not confirm the interactions and it does not advance the functional significance of the putative interactions.

It is not clear whether the altered levels of putative interactors found in the tobacco users is caused by the increased b2 and a4 levels (more physical binding sites) or by altered activity resulting from the increased cholinergic tone. It is also possible that tobacco users have altered levels of other nAChRs such as a7 whose activation may be the cause of the changes in the presynaptic proteins known to function in neuronal excitability.

Strengths:

1-) Unbiased proteomic approach; 14/33 putative interactors of b2 showed altered levels in postmortem tobacco users versus non-users; 2 of these correlated significantly with both tobacco use and mood disorders.

2-) Make excellent use of mouse mutants for shared changes with human b2*nAChR putative interactors. 8 proteins identified in both human and mouse cortex as shared b2*nAChR putative interactors, but did not vary with gene dosage

3-) use nicotine metabolite levels in postmortem human temporal cortex to evaluate tobacco usage

4-) Table 6 is a good inclusion for putative interactors often seen in control databases

Weaknesses:

1-) The mouse b2*nAChR co-precipitated proteins were not significantly altered by nicotine exposure after correcting for b2 abundance, as done for the human data, making it difficult to conclude the significance of the changes found in the human studies. Are the changed levels in humans caused by nicotine or by other factors such as altered activity in the cortical circuits.

2-) 14-3-3 zeta co-precipitated with human b2 but not mouse. What is the explanation for this difference?

3-) Cross linkers were used to stabilize labile interactions prior to detergent extraction but this could cause co-precipitation of neighboring proteins. It is important to discuss the size of the cross-linker and how it may alter the results. Some of the co-precipitated proteins are glial and others are presynaptic active zone and synaptic vesicle proteins, establishing the importance for testing the interactions by independent biochemical assays, without the cross-linker. Some statements (p.16, last sentence of 1st paragraph) should be modified "stable receptor interactions that survive tissue solubilization are with functional b2*nAChRs resident in the plasma membrane of neuronal synapses". Most of this (underlined parts) was not demonstrated. Similarly, last paragraph of discussion needs to be modified for same reasons.

4-) they conclude that the b2*nAChRs are presynaptic based on putative interactions with presynaptic neurotransmitter release proteins but they cannot rule out intracellular distribution of the cross-linked co-precipitated proteins and a postsynaptic localization of surface b2*nAChRs.

5-) need to show p values and state statistic tests used in Figure legends.

6-) In Fig. 3, why does the het show a greater increase in b2 levels with nicotine than the wild type? Fig. 3 legend has a typo, a2 is a4.

7-) Why were only female mice used for this study?

8-) Were protease inhibitors added during the homogenization and cross-linking?

Reviewer 2.

This manuscript reports on proteins associated with nicotinic acetylcholine receptors (nAChRs) identified by immunoprecipitation of the receptors followed by quantitative proteomic analysis of the associated components. The major focus of the report is to identify changes in the nAChR-associated proteasome caused by extended nicotine consumption/exposure. They examine these changes in mice subjected to nicotine, and in humans judged to have been smokers based on cotinine content in the postmortem tissue. Large numbers of components are found to be associated with nAChRs and, strikingly, many are altered by nicotine exposure. A substantial number of the affected components are common to mice and humans.

These are important findings both in terms of basic science and from a biomedical perspective, given the impact of smoking on human health. The results and conclusions appear quite solid. Previous work has demonstrated the ability of the antibody to capture the relevant nAChR subtypes for immunoprecipitation. And in the present studies the authors compare the levels of associated components captured by the antibody in mice of different genotype, i.e wild-type, heterozygous, &amp; knockouts for the relevant nAChR gene(s). This provides confidence that the associated component is indeed associated with the receptor, rather than being non-specifically associated with the antibody complex.

The one non-trivial concern is that it apparently was not possible to demonstrate in mice that the nicotine-induced changes in the nAChR-associated components were in fact also genotype-specific, i.e. that the increased amounts resulting from a nicotine history were seen in wild-type but not in knockouts, etc. The explanation offered appears to be that the numbers of animals (or animal pools) analyzed were too small to provide sufficiently small error bars. This is potentially a problem because one wants to be sure that not only are the components associated with the nAChR (and therefore immunoprecipitated by the antibody) but also that the increases seen after nicotine treatment reflect the same connection, rather than being the results of non-specific entrapment by the antibody complex. This may be a bit of a long-shot concern, but the authors may well want to address it more directly in the Discussion.
